# Effect of High Fructose Corn Syrup and Citric Acid in Broiler Diets on Performance, Gut pH, Immunity, Antioxidant Status and Some Blood Parameters

**DOI:** 10.1002/vms3.70436

**Published:** 2025-06-01

**Authors:** Gökhan Şen, Ali Karapinar, Mehmet Naci Oktay, Miyase Çinar, Mehmet Başalan

**Affiliations:** ^1^ Department of Animal Nutrition and Nutritional Diseases Faculty of Veterinary Medicine Kırıkkale University Kirikkale Turkey; ^2^ Department of Animal Breeding and Husbandry Faculty of Veterinary Medicine Kırıkkale University Kirikkale Turkey; ^3^ Department of Biochemistry Faculty of Veterinary Medicine Kırıkkale University Kirikkale Turkey

**Keywords:** blood parameters, broiler, citric acid, fructose, performance, syrup

## Abstract

The objective of this study was to determine the effect of high fructose corn syrup (HFCS) and citric acid (CA) in broiler diets on performance, gut pH, immunity, antioxidant status and some blood parameters. A total of 120 Ross 308 chicks at the age of 0 days were divided into 4 groups with 3 subgroups of 10 chicks each: control (CON), 50 mg/kg HFCS, 30 mg/kg CA and 50 mg/kg HFCS + 30 mg/kg CA (HFCS + CA). It was determined that body weight gain (BWG) was highest in the CA group and was similar to the CON group, whereas it was lower in the HFCS + CA group than other groups (*p* < 0.05). The feed conversion rates of the CA and the CON groups were similar and lower than the others (*p* < 0.05). Carcass weights and ratios increased in the CA group (*p* < 0.05). There were significant differences between the weights of liver and pancreas of groups (*p* < 0.05). Serum high‐density lipoprotein (HDL)‐CHO and GLU levels increased in the HFCS + CA group (*p* < 0.05). Serum total cholesterol (TCHO) level in the HFCS group was similar to the CON group and lower than the other groups (*p* < 0.05). Serum tumour necrosis factor‐α (TNF‐α) and interleukin‐1β (IL‐1β) values were determined to be highest in the HFCS group and different from the CON group (*p* < 0.05). It was observed that HFCS negatively affected performance, whereas CA positively affected. HFCS + CA increased serum TCHO and HDL‐CHO levels and CA led to an increasing trend in total antioxidant status (TAS) level. Although HFCS had a positive effect on TNF‐α and IL‐1β, similar of immunoglobulin G (IgG) and immunoglobulin M (IgM) levels between groups was observed.

## Graphical abstract

Although HFCS negatively affected performance both alone and when mixed with citric acid, citric acid alone positively affects performance. In addition, HFCS together with citric acid increased serum TCHO and HDL‐CHO levels. Although HFCS and citric acid do not have antioxidant effects as both mixtures and alone, it has been determined that citric acid would increase TAS levels. Serum TNF‐α and IL‐1β values increased with HFCS, but the additives had no effect on IgG and IgM levels.

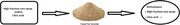

## 1 Introduction

Sugars are important energy sources for the body. The main components of dietary sugars are glucose and fructose. These components, which are mostly found in equal proportions, can also be found alone (Kolderup and Svihus 2015). Although both glucose and fructose are monosaccharides with identical formulas, their metabolism pathways are different (Taskinen et al. 2019). Glucose and fructose also differ from each other in terms of sweetness. Fructose is two times sweeter than glucose. The most used commercial form of fructose is high fructose corn syrup (HFCS) (Aşıcı et al. 2020).

Broiler farming aims to produce more meat with less feed. Studies conducted with fructose diets determined that body weight gain (BWG) increased (Simoyi and Klandorf 2003; Bocarsly et al. 2010). However, due to the increasing amount of fructose or HFCS in the diet, the tendency to metabolic syndrome, which includes diseases such as excessive fat in the internal organs, abdominal obesity, insulin resistance and hyperinsulinemia, increases (Ferder et al. 2010). Although the use of fructose‐containing fruit by‐products has become increasingly widespread in poultry farming in recent years, the effect of fructose on broiler metabolism is not yet fully known. Studies on this subject in poultry are limited in number (Altuğ et al. 2024; Ouchi et al. 2023; Şen et al. 2024).

Antibiotics, produced naturally by fungi and bacteria or synthetically, are used to treat and prevent infections in humans and animals by inhibiting or destroying bacteria. However, intensive antibiotic use increases the development of antibiotic resistance, and residues in feed and environment threaten human and animal health. Therefore, alternatives need to be developed to stop the increase of resistant bacteria and prevent the spread of infectious diseases (Mehdi et al. 2018). One of the various antibiotic alternatives is acidifiers. Organic acids such as citric, benzoic, fumaric and their salts can act as acidifiers. Each of the acidifiers has different levels of antibacterial effect. Acidifiers have three different modes of action: reduction of pathogens, suppression of inflammatory, regulation of pancreatic secretions and mucosal structure (Rahman et al. 2022). Organic acids are additives with properties similar to antibiotics that improve animal health and productivity by contributing significantly to intestinal health and development (Chukwudi et al. 2025). It has been shown that both the deterioration in intestinal morphology in infected broilers can be repaired and the number of pathogens in the intestinal content can be reduced with humic acid (Saleh et al. 2022).

Citric acid (CA) occurs naturally in citrus fruits, which is associated with the function of reducing oxidative damage in cells. It is artificially produced in the food industry, used for preservatives, sweeteners and acidifiers. CA has numerous applications for drug development in both the food and the pharmaceutical industries. Animal and clinical studies revealed that CA possesses antioxidant and anti‐inflammatory effects in the cell, can improve the immune system and protects the liver and brain. It can help maintain redox homeostasis and reduce lipid peroxidation of the cell (Singh et al. 2022). Many studies have been conducted to evaluate the effects of CA on performance, immune response and antioxidant status in poultry (Santana et al. 2019; Fikry et al. 2021; Krauze et al. 2021; Xue et al. 2023). Additionally, there are also studies showing that CA corrects glucose metabolism disorder, improves sugar utilization, increases insulin sensitivity and improves blood lipid levels in a hyperlipidemic state (Wang MiaoYing et al. 2019; Yadikar et al. 2022). However, no studies have analysed the effects of CA on growth performance, biochemical parameters, oxidative stress and immune response in broilers exposed to high fructose.

The aim of this study was to evaluate the effects of HFCS and CA added to broiler diets on performance, gut pH, immunity, antioxidant status and some blood parameters.

## 2 Material Method

The experiment was carried out with 120 1‐day‐old Ross 308 broiler chicks of both sexes for 42 days. The chicks were divided into four main groups, each with three subgroups. There was no statistical difference between the groups when the chicks were divided into groups (*p* = 0.565). The groups were as follows; (1) control consuming the feed containing no HFCS or CA (CON), (2) HFCS consuming the feed containing 50 mg/kg HFCS, (3) CA consuming the feed containing 30 mg/kg CA and (4) HFCS + CA consuming the feed containing 50 mg/kg HFCS + 30 mg/kg CA. For this purpose, 42% HFCS, which commonly used in solid foods (Parker et al. 2010) was used. Commercially available liquid HFCS (SUNAR, Türkiye) and powdered CA (BAKON, Türkiye) were mixed with powder form of other diet ingredients. The doses of HFCS (Şen et al. 2024) and CA (Nourmohammadi and Afzali 2013) used have been determined according to the previous studies. The chicks were placed in compartments with an area of 1 m^2^, with 10 chicks in each subgroup. The environment was heated with infrared lamps and electric heaters, starting with a temperature of 35 °C, and was gradually decreased as stated in the Ross broiler handbook (Anonymous 2014). Ambient lighting is provided 24 h a day with LED enlightening. Chicks were provided ad libitum access to water through chick drinkers for the first week and then through nipple drinkers. Feed was provided ad libitum in chick feeders for the first week of the study and then in hanging feeders. The chicks were fed on a broiler starter diet from 1 to 21 days. During this 21‐day period of the study, diets were presented in crumble form. Finisher diets from days 21 to 42 are offered in pellet form. The ingredients and chemical compositions of the diets used during the study are given in Table 1. The chicks were weighed weekly, and their average BWG was determined. On the days of body weight weighing, the remaining feeds in the feeders were also weighed, and the daily average feed consumptions were determined. Feed conversion ratios were calculated by dividing the average body weights by the average feed consumption.

**TABLE 1:** Ingredients and chemical compositions of experimental diets.

	CON		HFCS		CA		HFCS + CA	
Ingredients	Starter (%)	Grower (%)	Starter (%)	Grower (%)	Starter (%)	Grower (%)	Starter (%)	Grower (%)
Corn	46.25	48.50	47.05	48.85	43.00	50.00	43.75	47.50
Soybean meal (48% CP)	36.00	32.70	38.30	35.00	37.20	32.95	39.40	34.70
Wheat bran	9.60	8.70	2.00	1.60	7.25	4.00	—	—
HFCS	—	—	5.00	5.00	—	—	5.00	5.00
Citric acid	—	—	—	—	3.00	3.00	3.00	3.00
Vegetable oil	3.5	6.00	2.90	5.45	5.00	6.00	4.35	5.75
Dicalcium phosphate	2.5	2.00	2.50	2.00	2.50	2.10	2.35	2.10
Limestone	0.90	0.90	1.00	0.90	0.80	0.80	0.85	0.80
dl‐Methionine	0.40	0.35	0.40	0.35	0.40	0.30	0.40	0.30
l‐Lysine hydrochloride	0.25	0.25	0.25	0.25	0.25	0.15	0.20	0.15
l‐Threonine	0.10	0.10	0.10	0.10	0.10	0.20	0.20	0.20
Salt	0.40	0.40	0.40	0.40	0.40	0.40	0.40	0.40
Vitamin‐mineral premix^a^	0.10	0.10	0.10	0.10	0.10	0.10	0.10	0.10
**Chemical composition**								
Crude protein (g/kg)	230	210	230	210	230	210	230	210
Methionine + Cystine (g/kg)	11.2	9.9	11.1	9.8	11.1	9.6	11.0	9.5
Lysine (g/kg)	14.4	12.5	14.7	12.8	14.5	12.5	14.4	12.7
Treonin (g/kg)	9.5	8.5	9.6	8.5	9.6	9.8	10.6	9.8
Calcium (g/kg)	10.5	9.3	10.9	9.4	10.2	9.1	10.0	9.1
Phosphore (g/kg)	5.3	4.6	5.3	4.6	5.3	4.6	5.0	4.5
Sodium (g/kg)	1.8	1.7	1.8	1.7	1.8	1.8	1.7	1.7
ME (kcal/kg)	3027	3160	3027	3157	3026	3161	3027	3162

Abbreviations: CA, citric acid; CON, control; CP, crude protein; HFCS, high fructose corn syrup; HFCS + CA, high fructose corn syrup + citric acid; ME, metabolizable energy.

^a^ Vitamin A: 10,000.000 IU/kg; Vitamin D3: 3500.000 IU/kg; Vitamin E: 50,000 mg/kg; Vitamin K3: 2500 mg/kg; Vitamin B1: 2500 mg/kg; Vitamin B_2_: 6000 mg/kg; Vitamin B_3_: 50,000 mg/kg; Vitamin B_5_: 15,000 mg/kg; Vitamin B_6_: 3500 mg/kg; Vitamin B_12_: 15 mg/kg; Folic Acid: 1500 mg/kg; Biotin: 200 mg/kg; Manganese Sulphate: 100,000 mg/kg; Iron Sulphate: 25,000 mg/kg: Zinc Sulphate: 100,000 mg/kg; Copper Sulphate: 15,000 mg/kg; Iodine: 1550 mg/kg; Selenium: 450 mg/kg.

At the end of study, it was selected 36 chickens in total, 9 from each group. Blood was taken from these selected chickens via the jugular vein. Chickens whose blood was taken were sacrificed after blood collection processing. The carcass weights of the sacrificed animals were weighed after the head, feathers, legs and internal organs were removed. Carcass and visceral ratios were calculated by proportioning the obtained carcass or visceral weights to the live weight. Cecum content was also collected for pH measurement, whereas internal organs were removed.

The collected blood samples were centrifuged at 4°C and 1600 × *g* for 10 min, and plasma and serum were separated. Plasma and serum were stored at −80°C until analysis. Serum high‐density lipoprotein (HDL‐CHO), low‐density lipoprotein (LDL‐CHO), triglyceride (TG), total cholesterol (TCHO) and glucose (GLU) levels were determined by using an autoanalyzer (Mindray BS‐400). Serum insulin levels were measured with commercial ELISA kits (BT‐Lab/EA0012Ch) specific for chicken by using an ELISA device (Romer, BIO‐TEK EL X 800), according to the manufacturer's instructions.

Plasma malondialdehyde (MDA) concentrations were determined according to the procedure described for the thiobarbituric acid (TBA) reactive substances (TBARS) using the method of Buege and Aust (1978). The method is based on the formation of a pink colour under the acidic condition upon the reaction of MDA and TBA. The absorbance of the reaction product between MDA and TBA was measured spectrophotometrically at 536 nm. The data were expressed as µmol/L. Total oxidant status (TOS) was measured in plasma using commercially available kits (Rel Assay, Turkey) according to Erel (2005). TOS results were expressed as micromolar hydrogen peroxide equivalent per litre (µmol H_2_O_2_ Eq/L). Total antioxidant status (TAS) levels measured in plasma using commercially available kits (Rel Assay, Turkey) according to Erel (2004). The TAS results were expressed as micromolar Trolox equivalent per litre. The oxidative stress index (OSI) was calculated with the ratio of TOS to TAS. For this, the resulting unit of TAS was converted to µmol/L, and the OSI value was calculated according to the following:

OSI (arbitrary unit) = TOS (µmol H_2_O_2_ equivalent/L)/total antioxidant capacity (TAC) (µmol Trolox equivalent/L) (Harma et al. 2003; Kösecik et al. 2005; Yumru et al. 2009).

Levels of tumour necrosis factor‐α (TNF‐α) (BT Lab/EA0010Ch), interleukin‐1β (IL‐1β) (BT Lab/EA0003Ch), and interleukin‐6 (IL‐6) (BT Lab/EA0004Ch) were measured in serum with commercial ELISA kits specific for chicken by using an ELISA device (Romer, BIO‐TEK EL X 800), according to the manufacturer's instructions. Levels of plasma immunoglobulin G (IgG) and immunoglobulin M (IgM) were measured with commercial ELISA kits specific for chicken by using a microplate reader (Thermo Scientific Multiskan Go).

All data were analysed using the one‐way analysis of variance (ANOVA) using the ‘SPSS 15.0 for Windows’. Descriptive statistics were expressed as means and standard errors. Mean values were considered significantly different at *p* < 0.05. The differences between the groups were determined by Duncan's multiple range tests.

## 3 Results

The average BWGs of experiment groups are given in Table 2. Accordingly, the BWGs of the groups showed significant differences in every week of the experiment except the sixth week (*p* < 0.05). The BWGs of the HFCS + CA group were lower than the other groups (*p* < 0.05). Similar results are seen in the 3‐week and overall periods. However, in the second half of the study and the overall period, the BWGs of the CA group were similar to the CON and higher than the other groups (*p* < 0.05).

**TABLE 2:** Effects of HFCS and citric acid alone or in combination on average body weight gains in broilers, g.

Days	CON	HFCS	CA	HFCS + CA	*p*
7	146.75 ± 4.06^b^	138.05 ± 1.01^b^	137.70 ± 2.15^b^	110.03 ± 2.60^a^	<0.001
14	272.95 ± 19.93^b^	282.35 ± 12.55^b^	303.22 ± 8.07^b^	200.08 ± 8.18^a^	0.003
21	493.97 ± 45.29^b^	453.23 ± 5.46^b^	521.92 ± 3.20^b^	302.18 ± 12.32^a^	0.001
28	522.44 ± 35.77^b^	544.56 ± 19.14^bc^	606.67 ± 10.09^c^	430.00 ± 10.44^a^	0.003
35	695.33 ± 57.92^b^	539.11 ± 61.89^a^	738.89 ± 37.44^b^	506.67 ± 8.65^a^	0.019
42	537.67 ± 37.42	436.67 ± 38.44	486.96 ± 109.97	491.89 ± 48.88	0.762
1–21	913.67 ± 58.32^b^	873.63 ± 17.98^b^	962.83 ± 7.54^b^	612.30 ± 22.79^a^	<0.001
21–42	1755.44 ± 127.74^bc^	1520.33 ± 30.75^ab^	1832.52 ± 100.47^c^	1428.56 ± 61.00^a^	0.036
1–42	2669.12 ± 159.22^bc^	2393.97 ± 22.56^b^	2795.35 ± 93.72^c^	2040.86 ± 78.33^a^	0.003

*Note*: The mean values with different letters in the same row are significantly different (*p* < 0.05). Values are given as mean + standard error.

Abbreviations: CA, citric acid; CON, control; HFCS, high fructose corn syrup; HFCS + CA, high fructose corn syrup + citric acid.

The average feed consumptions of experiment groups are shown in Table 3. Generally, average feed consumption amounts of the HFCS + CA group were also lower than the other groups in weekly periods and in the first half of experiment (*p* < 0.05). The average feed consumption of the groups in the second half of the experiment and the overall period was similar (*p* > 0.05).

**TABLE 3:** Effects of HFCS and citric acid alone or in combination on average feed consumptions in broilers, g.

Days	CON	HFCS	CA	HFCS + CA	*p*
7	151.76 ± 2.77^b^	153.52 ± 4.51^b^	137.40 ± 2.76^a^	139.12 ± 2.54^a^	0.014
14	337.02 ± 14.67^b^	352.12 ± 11.27^b^	348.68 ± 7.96^b^	283.27 ± 11.16^a^	0.010
21	563.34 ± 45.39^b^	545.27 ± 7.66^b^	573.47 ± 3.22^b^	459.88 ± 15.24^a^	0.039
28	769.67 ± 46.62^ab^	821.27 ± 18.83^b^	844.00 ± 5.74^b^	710.50 ± 4.86^a^	0.025
35	1054.23 ± 76.57^b^	1019.80 ± 23.50^ab^	1105.22 ± 15.37^b^	905.23 ± 10.02^a^	0.045
42	1031.97 ± 72.13	1036.70 ± 10.51	1085.46 ± 47.04	1012.90 ± 39.95	0.745
1–21	1052.12 ± 56.13^b^	1050.90 ± 22.06^b^	1059.55 ± 12.97^b^	882.27 ± 28.48^a^	0.016
21–42	2855.87 ± 189.86	2877.77 ± 21.88	3034.69 ± 53.73	2628.63 ± 53.80	0.121
1–42	3907.99 ± 238.58	3928.67 ± 43.41	4094.24 ± 49.21	3510.90 ± 80.23	0.065

*Note*: The mean values with different letters in the same row are significantly different (*p* < 0.05). Values are given as mean + standard error.

Abbreviations: CA, citric acid; CON, control; HFCS, high fructose corn syrup; HFCS + CA, high fructose corn syrup + citric acid.

Feed conversion rates of experiment groups are seen in Table 4. In the first weeks of the experiment, the feed conversion rates of the HFCS + CA group were higher than the other groups (*p* < 0.05). In the following weeks, the feed conversion rates of the groups were statistically similar (*p* > 0.05). When 3‐week and overall periods were examined, the groups containing fructose were higher than the others (*p* < 0.05). However, in the first half period, the HFCS + CA group was higher than the HFCS group (*p* < 0.05).

**TABLE 4:** Effects of HFCS and citric acid alone or in combination on feed conversion rates in broilers, g/g.

Days	CON	HFCS	CA	HFCS + CA	*p*
7	1.03 ± 0.01^a^	1.11 ± 0.03^b^	1.00 ± 0.02^a^	1.27 ± 0.02^c^	<0.001
14	1.24 ± 0.04^a^	1.25 ± 0.02^a^	1.15 ± 0.01^a^	1.42 ± 0.04^b^	0.002
21	1.14 ± 0.04^ab^	1.20 ± 0.00^b^	1.10 ± 0.01^a^	1.52 ± 0.01^c^	<0.001
28	1.48 ± 0.10	1.51 ± 0.02	1.39 ± 0.01	1.65 ± 0.04	0.053
35	1.52 ± 0.03	1.94 ± 0.22	1.50 ± 0.06	1.79 ± 0.01	0.071
42	1.92 ± 0.01	2.41 ± 0.21	2.43 ± 0.47	2.08 ± 0.12	0.482
1–21	1.15 ± 0.02^b^	1.20 ± 0.00^b^	1.10 ± 0.01^a^	1.44 ± 0.02^c^	<0.001
21–42	1.63 ± 0.04^a^	1.89 ± 0.05^b^	1.66 ± 0.06^a^	1.84 ± 0.04^b^	0.011
1–42	1.46 ± 0.01^a^	1.64 ± 0.03^b^	1.47 ± 0.04^a^	1.72 ± 0.03^b^	<0.001

*Note*: The mean values with different letters in the same row are significantly different (*p* < 0.05). Values are given as mean + standard error.

Abbreviations: CA, citric acid; CON, control; HFCS, high fructose corn syrup; HFCS + CA, high fructose corn syrup + citric acid.

Carcass yields, visceral weights and cecum pH values of experiment groups are seen in Table 5. There was a significant difference between carcass weights of all groups (*p* < 0.05). The highest carcass weight was in the CA group, and the lowest was in the HFCS + CA group. The carcass weight of the HFCS group was lower than that of the CON group (*p* < 0.05). There was also a significant difference between the carcass ratios of the groups (*p* < 0.05). The highest carcass ratio was in the CA group, whereas the lowest carcass ratio was in the HFCS + CA group. CON and HFCS groups were similar (*p* > 0.05).

**TABLE 5:** Effects of HFCS and citric acid alone or in combination on carcass yields, visceral rates and cecum pH values in broilers.

Parameters	CON	HFCS	CA	HFCS + CA	*p*
Carcass (g)	2207.56 ± 45.30^c^	2019.67 ± 44.50^b^	2335.00 ± 32.85^d^	1607.56 ± 51.12^a^	<0.001
Carcass rate (%)	77.08 ± 0.58^b^	76.67 ± 0.66^b^	79.11 ± 0.69^c^	73.89 ± 0.49^a^	<0.001
Liver (%)	1.69 ± 0.05	1.65 ± 0.08	1.73 ± 0.05	1.91 ± 0.10	0.093
Spleen (%)	0.09 ± 0.01^a^	0.09 ± 0.01^a^	0.07 ± 0.00^a^	0.13 ± 0.01^b^	0.01
Pancreas (%)	0.16 ± 0.01	0.14 ± 0.01	0.14 ± 0.01	0.15 ± 0.01	0.298
Heart (%)	0.36 ± 0.02^a^	0.39 ± 0.02^a^	0.34 ± 0.01^a^	0.51 ± 0.02^b^	<0.001
Bursa fabricius (%)	0.19 ± 0.02	0.16 ± 0.03	0.12 ± 0.02	0.18 ± 0.02	0.118
Cecum pH	7.47 ± 0.12	7.40 ± 0.09	7.62 ± 0.09	7.45 ± 0.06	0.368

*Note*: The mean values with different letters in the same row are significantly different (*p* < 0.05). Values are given as mean + standard error.

Abbreviations: CA, citric acid; CON, control; HFCS, high fructose corn syrup; HFCS + CA, high fructose corn syrup + citric acid.

There was no significant difference between the liver ratios of groups (*p* > 0.05). The highest liver ratio was in the HFCS + CA group, and the lowest was in the HFCS group. There was significant difference between the spleen ratios of groups (*p* < 0.05). Although the spleen ratios of the HFCS and CA groups were similar to the CON group (*p* > 0.05), the spleen ratio of the HFCS + CA group was significantly higher than the others (*p* < 0.05). There was no significant difference between groups’ pancreas ratios (*p* > 0.05). The highest pancreas ratio was in the CON group, whereas the lowest pancreas ratio was in the HFCS and CA groups. The difference in heart rates between the groups was significant (*p* < 0.05). Although the heart ratios of the HFCS and CA groups were similar to the CON group (*p* > 0.05), the heart ratio of the HFCS + CA group was significantly higher than the others (*p* < 0.05). Bursa Fabricius ratios were similar between the groups (*p* > 0.05). Moreover, there was no significant difference between groups’ cecum pH values (*p* > 0.05).

The effects of dietary HFCS and CA supplementation separately or in combination on some biochemical parameters were given in Table 6. Serum TG, LDL‐CHO and insulin levels of groups were not statistically significant (*p* > 0.05). Glucose, HDL‐CHO and TCHO levels of groups were significantly different (*p* < 0.05). HDL‐CHO level of the HFCS + CA group was higher than others (*p* < 0.05). TCHO levels of the CA and HFCS + CA groups were similar (*p* > 0.05) and higher than the CON and the HFCS groups (*p* < 0.05). The GLU levels of the CON and HFCS groups were similar (*p* > 0.05) and higher than the CA group (*p* < 0.05). The GLU level of the HFCS + CA group was higher than other groups (*p* < 0.05).

**TABLE 6:** Effects of HFCS and citric acid alone or in combination on some serum biochemical parameters in broilers.

Parameters	CON	HFCS	CA	HFCS + CA	*p*
Insulin (μIU/mL)	4.27 ± 0.39	3.65 ± 0.07	3.62 ± 0.04	3.69 ± 0.07	0.104
LDL‐CHO (mg/dL)	29.22 ± 3.39	31.75 ± 3.56	37.89 ± 2.48	30.00 ± 2.04	0.159
HDL‐CHO (mg/dL)	90.67 ± 4.21^a^	82.81 ± 3.89^a^	92.12 ± 2.64^a^	108.68 ± 5.68^b^	0.002
TG (mg/dL)	19.78 ± 1.64	21.75 ± 2.77	22.00 ± 1.66	23.75 ± 2.27	0.614
TCHO (mg/dL)	133.00 ± 7.14^a^	127.13 ± 8.07^a^	153.44 ± 5.85^b^	160.75 ± 5.85^b^	0.004
GLU (mg/dL)	165.33 ± 6.65^b^	163.13 ± 2.28^b^	151.44 ± 4.22^a^	176.38 ± 4.48^c^	<0.001

*Note*: The mean values with different letters in the same row are significantly different (*p* < 0.05). Values are given as mean + standard error.

Abbreviations: CA, citric acid; CON, control; GLU, glucose; HDL‐CHO, high‐density lipoprotein; HFCS, high fructose corn syrup; HFCS + CA, high fructose corn syrup + citric acid; LDL‐CHO, low‐density lipoprotein; TCHO, total cholesterol; TG, triglyceride.

The plasma MDA concentrations, TAS, TOS and OSI levels in broilers treated with HFCS and CA were given in Table 7. Accordingly, there is no statistically significant difference between the groups (*p* > 0.05). However, the CA group’ TAS level was higher than the other groups, and there is a tendency for a statistical difference in the TAS value (*p* > 0.05).

**TABLE 7:** Effects of HFCS and citric acid alone or in combination on oxidant and antioxidant parameter values in broilers.

Parameters	CON	HFCS	CA	HFCS + CA	*p*
MDA (µmol/L)	1.23 ± 0.02	1.21 ± 0.06	1.17 ± 0.01	1.19 ± 0.06	0.912
TAS (mmol/L)	1.73 ± 0.07	1.84 ± 0.07	2.07 ± 0.15	1.77 ± 0.05	0.068
TOS (µmol/L)	5.16 ± 0.68	5.02 ± 0.84	4.43 ± 0.47	4.18 ± 0.34	0.608
OSI	0.30 ± 0.04	0.28 ± 0.05	0.22 ± 0.03	0.24 ± 0.02	0.408

Abbreviations: CA, citric acid; CON, control; HFCS, high fructose corn syrup; HFCS + CA, high fructose corn syrup + citric acid; MDA, malondialdehyde; OSI, oxidative stress index; TAS, total antioxidant status; TOS, total oxidant status.

Cytokine levels of experiment groups are shown in Table 8. Although there was a statistically significant difference in the TNF‐α and IL‐1β values of the groups (*p* < 0.05), the difference between the IL‐6 values of the groups was insignificant (*p* > 0.05). Although the TNF‐α value of the HFCS + CA group was similar to other groups (*p* > 0.05), the TNF‐α value of the HFCS group was significantly higher than the CON and CA groups (*p* < 0.05). In comparison, the HFCS group was higher compared to other groups (*p* < 0.05), and the CA group was similar to the CON and HFCS + CA groups (*p* > 0.05) in terms of IL‐1β. Moreover, the HFCS + CA group was higher than the CON group (*p* < 0.05). HFCS and CA, separately and combined, had no statistically significant effect on immune parameters (*p* > 0.05).

**TABLE 8:** Effects of HFCS and citric acid alone or in combination on cytokine levels and immunity parameters in broilers.

Parameters	CON	HFCS	CA	HFCS + CA	*p*
TNF‐α (ng/L)	39.42 ± 5.62^a^	64.13 ± 9.79^b^	44.08 ± 3.20^a^	56.37 ± 6.36^ab^	0.046
IL‐1β (ng/L)	53.39 ± 4.03^a^	97.41 ± 8.57^c^	62.30 ± 4.86^ab^	75.78 ± 6.50^b^	<0.001
IL‐6 (ng/L)	140.91 ± 17.73	106.94 ± 17.60	126.84 ± 18.17	110.78 ± 16.32	0.506
IgG (ng/mL)	4.88 ± 0.02	4.90 ± 0.02	4.91 ± 0.01	4.86 ± 0.02	0.258
IgM (pg/mL)	5.92 ± 0.16	5.81 ± 0.37	6.24 ± 0.49	6.10 ± 0.33	0.843

*Note*: The mean values with different letters in the same row are significantly different (*p* < 0.05). Values are given as mean + standard error.

Abbreviations: CA, citric acid; CON, control; HFCS, high fructose corn syrup; HFCS + CA, high fructose corn syrup + citric acid; IgG, immunoglobulin G; IgM, immunoglobulin M; IL‐1β, interleukin‐1β; IL‐6, interleukin‐6; TNF‐α, tumour necrosis factor‐α.

## 4 Discussion

This study provides information about the effects of HFCS and CA on performance, immunity, antioxidant status and some blood parameters in chickens. Accordingly, it was observed that BWG was negatively affected in the HFCS + CA group. In the second 3‐week period and the overall period, whereas there was a significant difference between CA and HFCS groups, both were similar to the CON group. The feed consumption of the groups was similar in the second 3‐week and overall periods but was low in the HFCS + CA group in the first 3‐week period. Miles et al. (1987) stated that the particles of feed containing HFCS tend to adhere to each other, and this may be due to the change in the physical structure of the feed. There was no physical change due to particle adhesion in the feed used in the study. In overall period, although there is no difference in feed consumption between the groups and the feed conversion rate is higher than the CON group, it is seen that the BWG of the HFCS group is similar with the CON group. Although there is no statistical difference in HFCS supplemented feeding, more feed is consumed for per unit BWG. Glucose intake contributes to satiety because it affects insulin secretion, which increases leptin release. Unlike glucose, fructose does not trigger the normal satiety response because it does not acutely stimulate the release of leptin or insulin (Yılmaz and Yılmaz 2019). However, contrary to expectations, there was no significant difference between the insulin levels of the groups. HFCSs are available in 42%, 55% and 90% (Karaoğlu 2011). The fact that the expected effect on insulin is not observed may be because the HFCS used contains 42% fructose. It was determined that the BWG of the CA group in the second 3‐week period and the overall period, although it tends to increase, was similar to the CON group. Unlike the presented study, Khosravinia et al. (2015) showed that CA positively affected on performance. In the study conducted by Fik et al. (2021) with the addition of 0.5%, 1.0% and 1.5% doses of CA in broilers, BWG increased significantly compared to the control group in the second part of the study. The authors stated that this may be partly due to CA improving gut health. As a result of the study conducted by Asgar et al. (2013), it was stated that CA could be an alternative growth promoter compared to antibiotics. In studies comparing organic acids with antibiotics, it has been shown that performance can also be increased with organic acids (Bagal et al. 2016; Saleh et al. 2024). In the presented study, although CA did not affect the feed conversion ratio in the overall period, it was observed to have a positive effect in the first half of the study. Abou‐Ashour et al. (2021) concluded that feeding with 2% CA supplemented feeds positively affected feed conversion ratio both in 3‐week periods and in the overall period. As the reason for this increase, the authors attributed to the increased proteolysis with acidification and the metabolic roles of various organic acids in many metabolic pathways.

The negative effect on the live performance of the HFCS + CA group is also reflected in carcass weight and ratio. The carcass weight of the CA group, which had the highest body weight in the overall period, was significantly higher than the other groups, and a similar situation was observed in the carcass ratio. Bocarsly et al. (2010) stated that although increased body weight alone is not an indicator for obesity, the co‐occurrence of TG and fat accumulation in the body supports obesity. Their study with HFCS showed that the fat pads of the HFCS groups were heavier and their TG levels were higher than the control group. Although body fat levels were not examined in the current study, TG levels were similar between groups. Accordingly, it can be said that there is no obesity due to the effect of HFCS in the current study. The carcass ratio of the CA group was significantly higher than the other groups. Although the TG level, as an indicator of obesity, was not different between groups, the reason for this high carcass ratio may be muscle, not fat. As a matter of fact, adding CA to diets improves protein efficiency. This is due to the conversion of pepsinogen to pepsin, which increases pepsin activity and improves protein digestibility. In addition, peptides released by pepsin proteolysis also stimulate the release of hormones effective in protein digestion and absorption (Abou‐Ashour et al. 2021). It was determined that CA together with HFCS causes low body weight and carcass ratio.

The effect of HFCS and CA on internal organ ratios was determined to be significant in the spleen and heart. A significant increase in spleen ratio was observed in the HFCS + CA group compared to the other groups, and this increase may be due to the strengthening of the inflammatory response due to the synergistic effect of high fructose and CA. This result is also supported by the numerical increase in serum TNF‐α and IgM levels in the HFCS + CA group compared to the CON group. The significant increase in heart rate observed in the HFCS + CA group compared to the other groups may be causing metabolic stress of fructose due to the synergistic effect of high fructose and CA and the effects of CA on the cardiovascular system. In the present study, serum TCHO levels were significantly higher in the HFCS + CA and CA groups compared to the CON group, confirming the effect of CA on the cardiovascular system. Although the increase observed in liver rates was not statistically significant, the highest value observed in the HFCS + CA group can be explained by the de novo lipogenesis mechanism resulting from the hepatic metabolism of fructose (Örek and Mertoğlu 2020). In the presented study, the higher serum TG and TCHO levels in the HFCS + CA group compared to the other groups also support this result.

Fructose has the same chemical formula as glucose, but its metabolism is different. After digestion, in intestinal cells and hepatocytes can be convert fructose into glucose, glycogen, lactate and lipids. Hepatic de nova lipogenesis and lipotoxicity, oxidative stress and hyperuricemia have been proposed as responsible mechanisms for the adverse metabolic effects of fructose (Tappy and Lê 2010; Sievenpiper et al. 2012). Many sources are reporting that consuming one of the nutritive sweeteners containing high fructose or glucose/fructose will cause dyslipidemia (increased TG, TCHO and LDL‐CHO levels, decreased HDL), hyperglycemia and insulin resistance, which are indicators of metabolic syndrome in humans and animals (Elliott et al. 2002; Johnson et al. 2007; Abdullah et al. 2009; Gun et al. 2016; Hidayati et al. 2020). Miatello et al. (2005) found that there was no significant change in plasma TCHO and HDL‐CHO in rats given 10% fructose and 10 mg/kg resveratrol orally in drinking water for 45 days compared to the control group, and that although the increase in fasting plasma GLU levels in the fructose‐receiving groups was not statistically significant, the increase in plasma TG levels in these groups was significant. In a study (Abdullah et al. 2009), it was determined that in rats fed with fructose (0.3%) containing diet, serum TG and HDL‐CHO levels significantly increased in the fourth and eighth weeks compared to the control group, but LDL‐CHO levels did not change. The same study showed that serum GLU levels were within normal values for 8 weeks and no significant change was observed in both animal groups, and plasma insulin levels in the high‐fructose group were significantly higher than the control group. The increase in TG values is attributed to increased hepatic lipogenesis with fructose intake, excessive production of VLDL, and impaired peripheral catabolism (Busserolles et al. 2002; Elliott et al. 2002; Chong et al. 2007; Lowndes et al. 2014). From a mechanistic point of view, fructose is primarily absorbed in the intestine, transferred to the portal circulation and phosphorylated in the liver by fructokinase to fructose‐1‐phosphate. The latter molecule is converted to glycerol‐3‐phosphate, leading to the synthesis of glycerol, or it can be metabolized to acetyl‐CoA and incorporated into fatty acid via de novo lipogenesis. Fatty acids can combine with glycerol and form TG. This may be one of the main mechanisms of fructose‐induced hyperlipidemia (Abdullah et al. 2009). Gun et al. (2016) found that HFCS added to drinking water at a concentration of 30% for 6 weeks increased serum TG, TCHO, HDL‐CHO and LDL‐CHO levels compared to the control group. Şen et al. (2024) found that plasma TG, TCHO, HDL‐CHO and LDL‐CHO levels in broilers fed with low HFCS (50 mg/kg) and high HFCS (100 mg/kg) diets for 6 weeks were numerically lower than the control group but not statistically significant. Although the LDL‐CHO levels of the HFCS groups were similar, they were significantly lower than the control group. They stated that the lack of hyperlipidemia observed in the HFCS supplemented groups could be due to the use of broiler chickens, the net energy capacity of the ration, and the isocaloric adjustment of the diets given to the groups. In a study conducted on C57Bl/6 mice consuming water containing 55% fructose and 45% sucrose for 16 weeks (Kohli et al. 2010), it was found that there was no difference in plasma TG levels, but TCHO levels were high. Yıldırım (2012) reported that serum TG values were higher in rats consuming 10% and 20% high‐fructose corn syrup for 10 weeks compared to the control group, there was no significant change in TCHO values, and HDL‐CHO values were higher in rats consuming 10% fructose. Miatello et al. (2005) found that there was no significant change in plasma TCHO and HDL‐CHO in rats given 10% fructose and 10 mg/kg resveratrol orally in drinking water for 45 days compared to the control group, and that although the increase in fasting plasma glucose levels in the fructose‐receiving groups was not statistically significant, the increase in plasma TG levels in these groups was significant. In the presented study, it was observed that there was no significant change in plasma TCHO, HDL‐CHO levels in the HFCS groups compared to the control group, consistent with the studies conducted by Miatello et al. (2005) on rats, in LDL‐CHO levels consistent with the findings of Abdullah et al. (2009), and in TG levels consistent with the findings of Kohli et al. (2010). It has been stated that fructose feeding may reduce lipoprotein lipase activity and result in moderate hypercholesterolemia and hypertriglyceridemia (Abdullah et al. 2009).

CA is produced artificially in the food industries and is used for preservatives, sweeteners and acidifiers (Singh et al. 2022). It has been reported that serum TG, CHO, LDL‐CHO levels were lower and HDL‐CHO levels were higher in broilers receiving 0.8 mg/L CA with drinking water compared to the control group (Kassem et al. 2023). A study investigated of effects of 5, 10, 15 and 20 g/kg CA supplementation to Japanese quail diets by Fikry et al. (2021) showed that serum TCHO and LDL‐CHO concentrations significantly decreased. In contrast, HDL concentration significantly increased in groups supplemented with 10 or 15 g CA/kg compared to the control group. It has been reported that dietary CA supplementation of 2% to 3% in geese significantly reduced serum TCHO and LDL‐CHO levels compared to the control group. Dietary acidifiers have also been reported to significantly reduce blood cholesterol and total lipids (Kamal and Ragaa 2014). It has been reported that the beneficial role of organic acids in lowering total lipid and cholesterol may be due to their ability to reduce microbial intracellular pH (Abdel Fattah et al. 2008). It was determined by Coşar and Karslı (2023) that the addition of organic acids to broiler feeds did not change serum LDL‐CHO and TCHO levels, but TG and HDL‐CHO levels increased. Fouladi et al. (2018) reported that with the addition of organic acids such as acetic acid and lactic acid to the feed of female quails, serum HDL‐CHO and TCHO levels significantly increased, TG levels decreased and LDL‐CHO levels did not change. In the presented study, contrary to the findings of Fikry et al. (2021) and in parallel with the findings of Fouladi et al. (2018), it was found that serum TCHO and HDL‐CHO levels increased in the groups given CA compared to the control group. The different results obtained in lipid parameters in the studies suggest that more research is needed.

The fructose metabolism differs from glucose and occurs by an insulin‐independent mechanism (Tran et al. 2009). The relationship between fructose and insulin resistance and diabetes is still a matter of debate. It is known that fructose consumption does not trigger insulin release, unlike glucose, because the fructose transporter system GLUT5 is found in small amounts in the β cells of the pancreas (Curry 1989). Although this suggests that fructose is a recommendable nutrient for diabetic patients, some long‐term animal studies have shown that fructose consumption increases insulin resistance, impairs glucose tolerance and causes hyperinsulinemia (Kohli et al. 2010; Bagul et al. 2012). Kohli et al. (2010) stated that fructose‐consuming blood insulin and GLU levels increased, and insulin resistance developed. Studies with rats have shown that high fructose increased serum GLU and insulin levels (Bagul et al. 2012; Gun et al. 2016; Abdelkarem and Fadda 2017). On the other hand, Şen et al. (2024) reported that plasma GLU (*p* < 0.01) and insulin (*p* > 0.05) levels were lower in the HFCS groups compared to the control group in broilers fed with a low HFCS diet for 6 weeks, and that the decrease in GLU levels may be due to increased glucokinase activity and that short‐term or low‐rate fructose consumption may have a regulatory effect on blood GLU levels, and that long‐term exposure may increase blood GLU levels. In the presented study, in parallel with the study by Şen et al. (2024), plasma insulin and GLU levels were insignificantly lower in the HFCS group compared to the CON group. Although circulating glucose increases insulin secretion from the pancreas (Vilsbøll et al. 2003), fructose does this less effectively because there is no fructose transporter in the cells of the pancreas (Curry 1989; Sato et al. 1996).

A study on hyperlidemic rats reported that CA reduced blood GLU and insulin resistance index and increased insulin sensitivity (Yadikar et al. 2022). Mirderikvandi et al. (2019) reported negative effects on serum GLU in diets supplemented with organic acids in broiler. Elnagar and Abo EL‐Maaty (2017) and Solanki et al. (2020) reported that CA supplementation in ducks feeds did not affect serum GLU levels. In this study, it was found that serum GLU levels decreased in the CA group compared to the CON group as similar to the studies of Abou‐Ashour et al. (2021), who reported that 2% CA added to broiler feed reduced serum GLU levels in broilers. In the presented study, the significant decrease in serum GLU levels in the CA and HFCS + CA groups compared to the CON group may be due to the positive effect of CA on glucose metabolism, and the fact that insulin levels remained constant despite the decrease in serum GLU levels may be due to the increase in insulin sensitivity. The different results obtained on GLU and insulin in the studies suggest that more research is needed on this subject.

Oxidative stress plays an important role as a mediator of the damage produced by fructose metabolism (Kitagawa et al. 2020; Unsal et al. 2021). It was revealed by Pasko et al. (2010) that fructose (310 g/kg feed for 5 weeks) caused oxidative stress manifested by significant changes in MDA level and insignificant changes in enzymatic antioxidant potential in plasma of rats. In another study (Kostogrys and Pisulewski 2010), it was determined that plasma MDA concentrations significantly increased in rats whose diets were supplemented with fructose (63.2% fructose) for 21 days. It was stated that increased lipid peroxidation in fructose‐fed rats may be related to the high amount of glucose in the bloodstream, which may be due to increased free radical production due to glucose autoxidation and protein glycation. It has been reported that fructose feeding may induce free radical formation by downregulating of hexose monophosphate shunt enzymes that produce a reduced environment in the form of NADPH and NADH. They also stated that an increase in fructose catabolism may cause energy depletion in cells and make them more susceptible to peroxidation (Nandhini et al. 2005). Şen et al. (2024) showed that an increase in plasma TAS levels and a decrease in plasma TOS levels were significantly observed in the HFCS groups, and the OSI value was significantly lower in the low‐HFCS group compared to the control group and similar to the high‐HFCS group. In the presented study, consistent with the findings of Şen et al. (2024), an increase in plasma TAS levels and a decrease in MDA, TOS and OSI levels were observed in the HFCS group compared to the CON group. It was stated by Şen et al. (2024) that these results may be due to the fact that HFCS may have a reducing effect on oxidative damage in the short term and at the given dose (50 g/kg).

CAs have antioxidant properties in poultry. It has been reported that serum MDA levels were lower in broilers receiving 0.8 mg/L CA with drinking water compared to the control group (Kassem et al. 2023). In another study reported that 5, 10, 15 and 20 g/kg CA supplementation to Japanese quail diets increased TAC, GPx and SOD activities and decreased MDA concentrations (Fikry et al. 2021). It was found that plasma MDA concentration was significantly decreased in geese supplemented with 1.00% and 2.00% CA in their diets, but TAC, SOD, CAT and GPx activities were not affected (Xue et al. 2023). Researchers stated that dietary CA supplementation reduced lipid peroxidation, which may be due to decreased polymorphonuclear cell degranulation and weakened release of myeloperoxidase in poultry (Abdel‐Salam et al. 2014).

It has been stated that excessive fructose consumption is the main cause of obesity, diabetes and metabolic syndrome, and that these disorders are generally associated with immune system deterioration. Many clinical and animal studies have shown that excessive fructose intake leads to hepatic lipid accumulation and decreased insulin sensitivity, accompanied by a large increase in inflammatory factors. High dietary fructose levels have been shown to increase lipid and proinflammatory cytokine accumulation by regulating gut microbiota and metabolites. Excessive fructose intake has been suggested to disrupt the body's immune homeostasis by promoting immune cell metabolic regulation, changes in gut microbial community structure, and intestinal barrier permeability (Cheng et al. 2022). In this study, it was found that plasma TNF‐α and IL‐6 levels were higher compared to the control group as parallel to the study of Sivakumar et al. (2011), who reported that fructose added to rat feed increased plasma TNF‐α and IL‐6 levels compared to the control group. It has been reported that the increase in TNF‐α and IL‐6 in fructose‐fed rats may be related to nuclear factor kappa‐light chain enhancer of activated B cells (NF‐κB) activation, which is attributed to the increase in ROS levels and oxidative stress. NF‐κB activation increases the expression of many proinflammatory cytokines and stimulates the inflammatory cascade in fructose‐fed rats (Sivakumar et al. 2011). Fructose exhibits prooxidative and proinflammatory properties by inducing the formation of free radicals, reducing enzymatic and non‐enzymatic protection, inducing the production of proinflammatory cytokines and activating the NF‐κB (Khitan et al. 2013).

Organic acids can activate both specific and nonspecific immune responses by motivating macrophages, increasing cytokine production and increasing IgA, IgG and IgM concentrations in broiler chickens (Ebeid and Al‐Homidan 2022). It has been reported that the addition of 2.0% and 3.0% CA to the feed of ducklings increases serum IgA, IgM and IgG levels and can be safely used as natural growth promoters to improve the growth and immune response of ducklings (Elnagar and Abo EL‐Maaty 2017). Fikry et al. (2021) found that IgG level was higher in the groups receiving 5 and 10 g/kg CA than in the control group. In the presented study, no significant changes were observed in plasma IgG levels in the experimental groups compared to the control group. In IgM levels, numerical increases were observed in the CA‐added groups compared to the control group, consistent with the findings of Elnagar and Abo EL‐Maaty (2017).

In conclusion, it was determined that although HFCS negatively affected performance both alone and when mixed with CA, CA alone positively affects performance. In addition, HFCS together with CA increased serum TCHO and HDL‐CHO levels. Although HFCS and CA do not have antioxidant effects as both mixtures and alone, it has been determined that CA would increase TAS levels. Serum TNF‐α and IL‐1β values increased with HFCS, but the additives had no effect on IgG and IgM levels.

## Author Contributions

**Gökhan Şen**: investigation, methodology, funding acquisition, writing – original draft, writing – review and editing, project administration, formal analysis, validation, visualization. **Ali Karapinar**: investigation. **Mehmet Naci Oktay**: investigation. **Miyase Çinar**: writing – review and editing, writing – original draft, supervision, methodology. **Mehmet Başalan**: data curation, supervision.

## Ethics Statement

The experimental procedures were approved with the 2022‐06 meeting and decision number 36 by the Kırıkkale University Animal Experiments Local Ethics Committee.

## Conflicts of Interest

The authors declare no conflicts of interest.

## Data Availability

The data that support the findings of this study are available from the corresponding author upon reasonable request.
